# Nanoscale particles in technological processes of beneficiation

**DOI:** 10.3762/bjnano.5.53

**Published:** 2014-04-11

**Authors:** Sergey I Popel, Vitaly V Adushkin, Anatoly P Golub'

**Affiliations:** 1 Institute for Dynamics of Geospheres of the Russian Academy of Sciences, Leninsky pr. 38, bldg. 1, 119334 Moscow, Russia; 2 Moscow Institute of Physics & Technology, 141700 Dolgoprudny, Moscow region, Russia

**Keywords:** cavitation disintegration, gold ore, nano- and microparticles, polymineral and monomineral fractions

## Abstract

**Background:** Cavitation is a rather common and important effect in the processes of destruction of nano- and microscale particles in natural and technological processes. A possible cavitation disintegration of polymineral nano- and microparticles, which are placed into a liquid, as a result of the interaction of the particles with collapsed cavitation bubbles is considered. The emphasis is put on the cavitation processes on the interface between liquid and fine solid particles, which is suitable for the description of the real situations.

**Results:** The results are illustrated for the minerals that are most abundant in gold ore. The bubbles are generated by shock loading of the liquid heated to the boiling temperature. Possibilities of cavitation separation of nano- and microscale monomineral fractions from polymineral nano- and microparticles and of the use of cavitation for beneficiation are demonstrated.

**Conclusion:** The cavitation disintegration mechanism is important because the availability of high-grade deposits in the process of mining and production of noble metals is decreasing. This demands for an enhancement of the efficiency in developing low-grade deposits and in reprocessing ore dumps and tailings, which contain a certain amount of noble metals in the form of finely disseminated fractions. The cavitation processes occuring on the interface between liquid and fine solid particles are occasionally more effective than the bulk cavitation processes that were considered earlier.

## Introduction

At present, significant attention is being paid to the study of properties and processes of formation of mineral nano- and microsize particles [1–2]. The investigation of nanosize structures in nature can provide new information about the interstellar substance [3], deflation of the rock mass, etc. Nanosize components are the main structural elements in solid-state chemistry [4]. However, in many situations, physical processes such as cavitation are of paramount importance in the formation of nano- and microsize structures. For example, more than thirty years ago, Galimov [5] predicted the possibility of diamond synthesis through a cavitation process. In 2003, this possibility was confirmed in experiments [6]. The particles formed in the cavitation process were an aggregation of nanosize crystallites. The diamond crystals were 10–30 nm in size. The idea of the cavitation mechanism of diamond formation is the following [5]: A narrow canal cavity of varying cross section is formed as a result of the fast motion of fluid going up from the mantle to the surface of the Earth. When the canal cavity dilates or opens up, the pressure decreases, resulting in phase separation of the fluid, which stratifies into an essentially liquid phase and a gas phase existing in the form of gas bubbles. When the fluid goes into the narrowing of the canal cavity, the pressure is reestablished and the bubbles collapse. The pressure inside the collapsing bubbles, which are filled with carbon-containing gas is sufficient for the synthesis of diamonds.

The mechanism of cavitation melting was discussed in [7] while explaining the origin of microscopic globules found in cavities and fractures of vein quartz from mesothermal gold deposits. Adushkin et al. [8–10] developed an internally consistent theory that describes the formation of mineral nano- and microspherules through a cavitation mechanism that takes into account typical dimensions of cavitation bubbles and their evolution along with the dynamics of particle heating in cavitation bubbles. On the basis of this theory, the maximum dimensions of cavitation bubbles were estimated, as well as the size of globules generated due to the melting of particles of different mineral composition under cavitation effects in hydrothermal fluids. It was shown that the cavitation mechanism may bring about the formation of mineral and metallic nanospherules in hydrothermal fluids. The formation of nano- and microspherules with regard to the depth of host rock was also investigated.

In [11] the possible cavitation disintegration of polymineral microparticles placed into a liquid as a result of the interaction of particles with collapsing cavitation bubbles was shown for the minerals most abundant in gold ore. This disintegration mechanism is important because the availability of high-grade deposits in the process of mining and production of noble metals is decreasing. This demands for an enhancement of the efficiency in developing low-grade deposits and in reprocessing ore dumps and tailings, which contain a certain amount of noble metals in the form of finely disseminated fractions. The recovery of disseminated metals from fractions no more than 100 μm in size is a complicated problem, which can be solved with the use of the cavitation mechanism of disintegration of microscale particles.

**Figure 1:**
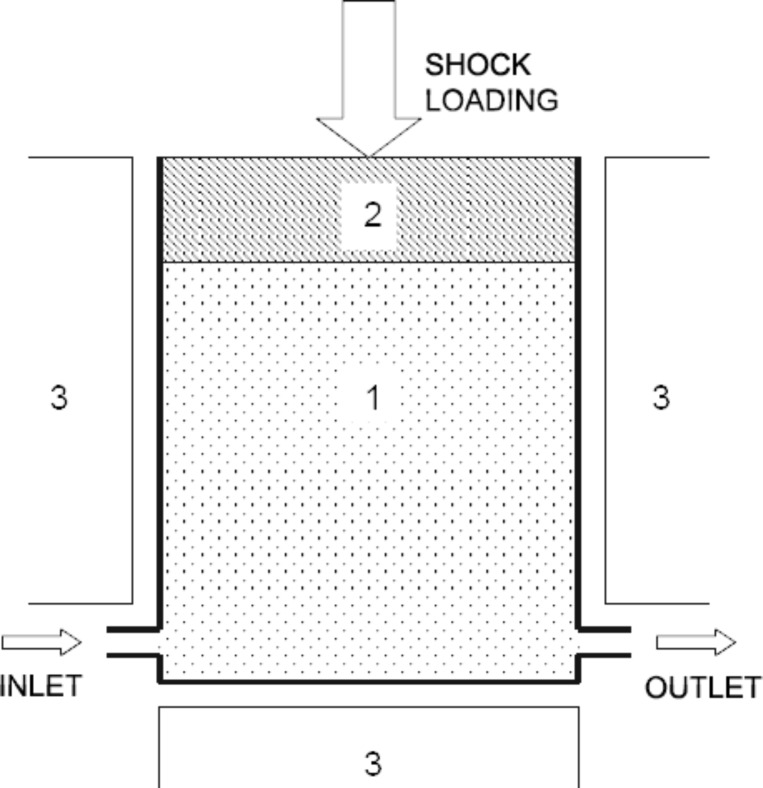
A conceptual design of a device for cavitation separation of gold-bearing particles. (1) Cavitation chamber with liquid (water) containing ore microparticles; (2) plunger providing shock loading (an abrupt rise in pressure in the chamber to 10–15 bar); (3) thermostat maintaining a temperature *T*_0_ = 373 K in the cavitation chamber.

The proposed method of cavitation disintegration is the following: Ore microparticles that contain finely disseminated fractions of noble metals are placed into a cavitation chamber (Figure 1) filled with a liquid (water) heated up to the boiling temperature. It is known that, in the process of heterogeneous boiling, the bubbles in a liquid nucleate and grow mostly on foreign inclusions: dust particles, roughnesses of the vessel walls, etc. Therefore, ore microparticles will serve as natural centers of cavitation bubble nucleation. If the pressure in the cavitation chamber rises abruptly, for instance, owing to shock loading by a plunger (press), the cavitation bubbles will collapse. Compression and collapse of a cavitation bubble is commonly accompanied by an abrupt local rise in temperature and pressure that can induce melting of an ore microparticle attached to the bubble and lead to its subsequent separation into monomineral fractions. The monomineral fractions obtained by cavitation melting may afterward be divided using standard techniques, e.g., gravity or chemical separation.

In [8–11] a one-dimensional spherically symmetric model, in which the nano- or microscale particle is placed in the center of the cavitation bubble is given. In general, this model does not describe the real situations when the bubbles are formed on foreign inclusions: dust particles, roughnesses of the vessel walls, etc. Here, we provide insights into the separation of polymineral microparticles into monomineral fractions in the process of interaction of the particles with collapsing cavitation bubbles. In contrast to [8–11], we propose the consideration of the cavitation processes on the interface between liquid and fine solid particles (or roughness of vessel wall), which is more suitable for the description of the real situations.

## Model

We numerically investigate the cavitation melting of mineral microparticles and the possibility of their destruction while using physical parameters of the minerals from gold ore. The mineral and chemical compositions of ores at large gold deposits, such as Cripple Creek (United States), Bagio (Philippines), and Pueblo Viejo (Dominican Republic), and at gold deposits in Russia are fairly complex [12], although the number of major minerals is relatively low. Native gold often occurs together with quartz and is always associated with iron, copper, antimony, lead, and zinc sulfides. Quartz, barite, and carbonates are the major gangue minerals. In addition to native gold, the primary ores contain pyrite, arsenopyrite, sphalerite, and galena. Several of the most abundant and important minerals of gold ore were chosen for the numerical analysis of the cavitation melting effect. Their physical parameters (density ρ, thermal diffusivity χ, and melting temperature *T*_m_) necessary for calculations are given in the Table 1.

**Table 1:** Physical parameters of the minerals from gold ore.

mineral	formula	ρ, g/cm^3^	χ, cm^2^/s	*T*_m_, K

gold	Au	19.3	1.3	1336
silver	Ag	10.5	1.73	1235
quartz	SiO_2_	2.14	0.042	1883
pyrite	FeS_2_	4.9	0.15	1461
galena	PbS	7.3	0.23	1387
calcite	Ca(CO_3_)	2.71	0.017	1885
stibnite	Sb_2_S_3_	4.6	0.145	819

The dynamics of a cavitation bubble in response to an abrupt rise of pressure *P*_0_ in the cavitation chamber due to shock loading was calculated with a numerical method by using a model, which has the main features described below. The cavitation processes on the interface between liquid and fine solid particles (or roughnesses of the vessel walls) result in an appearance of cavitation bubbles of mushroom-like form. The size of the bubbles, the time of their presence on the solid surface, the rate of detachment from the surface, etc. depend on local variations of wettability, roughness of the surface, its bending, and other random actions, which cannot be taken into account. Under the steady-state conditions the bubble is stable, the gas pressure inside the bubble is equalized by the external pressure (in the liquid surrounding the bubble). If the pressure in the liquid becomes larger than the gas pressure inside the bubble then the equilibrium is violated, the liquid presses forward to the solid surface, and leads to a collapse of the bubble. Pressure and temperature of the gas inside the bubble increase as the volume reduces. If the pressure in the liquid increases slowly and becomes higher than the pressure of saturated vapor (at the temperature equal to the temperature of liquid) then the bubble disappears. In case of a sufficiently rapid increase of the pressure, the vapor in the bubble does not have enough time to be condensed. The pressure inside the bubble increases so that the liquid head is restrained and the liquid itself retreats back. Rapid compression of the bubble results in very high magnitudes of density, pressure, and temperature of vapor, which are independent from the initial volume and geometric shape of the bubble. The motion of vapor inside the bubble remains subsonic. Gas dynamic processes dominate over those of thermal conductivity. Since the vapor temperature is significantly higher than that of the liquid and the phase equilibrium is violated, it is necessary to take into account the heat exchange between vapor and liquid due to phase transformation. Furthermore, imperfection of dense gas in the bubble may be an important factor in determining the dynamics of the bubble compression.

The dynamics of cavitation bubble was calculated under the following assumptions to simplify the problem: (1) The liquid, water, is incompressible with a density of ρ_l_ = 1 g/cm^3^ and the boiling temperature *T*_0_ = 373 K. (2) The cavitation bubbles do not interact with each other. The bubble shape is hemispherical with the center placed on the interface between liquid and fine solid particle. As the volume of the bubble changes, its geometric shape is maintained. (3) The bubble dynamics, the vapor motion, as well as liquid heating are described within the corresponding spherically symmetric model. The state of the vapor in the bubble is described by the van der Waals equation with the critical parameters *T*_cr_ = 647 K and *P*_cr_ = 225 bar. When calculating the gas dynamic processes inside the bubble, the heat exchange between the vapor and fine solid particles is neglected in comparison with the much more intensive (accompanied by a phase transition) heat exchange between the vapor and liquid. (4) The polymineral nature of the fine solid particle is taken into account in the following manner: The real geometrical shape of the particle is not considered, only its characteristic linear size (the diameter of the particle of nearly spherical shape or the thickness of the particle of some flat shape) and the characteristic linear dimension of its noble-metal core are introduced. Processes of heating and melting of the fine solid particle in its interaction with the bubbles are described within the framework of one-dimensional (plane) problem of thermal conductivity for the corresponding three-layer incompressible body, in which the noble metal is placed between two layers of mineral.

In this case, the dynamics of the bubble compression is described by the Rayleigh–Plesset equation [13]:

[1]
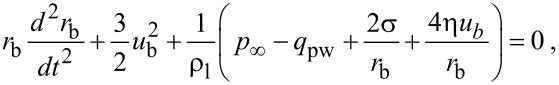


where *t* is time coordinate, *r*_b_ is the radius of the bubble, *u*_b_ is the speed of the bubble edge, *p*_∞_ is the pressure in the liquid far from the bubble, *q*_pw_ is the momentum flux density on the edge of the bubble from the side of the vapor, and *η* and *σ* are the viscosity and surface tension of the liquid, respectively. The set of equations of gas-dynamics, which is solved together with the van der Waals equation of state describes the vapor motion as well as the pressure, the temperature, and the internal energy inside the bubble. The change in the liquid temperature is determined by the set of thermal conductivity equations that, in particular, takes into account the heat exchange with the vapor. The liquid velocity is determined by the continuity equation for the incompressible liquid.

The set of gas-dynamics and thermal conductivity equations describing the vapor dynamics inside the bubble is:

[2]
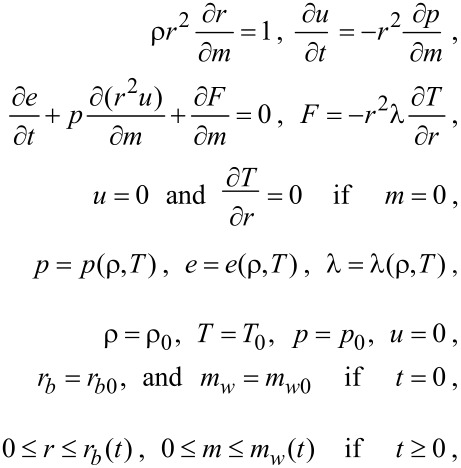


where *r* is the radius (Eulerian coordinate), *m* is Lagrangian (mass) coordinate, *m*_w_(*t*) is the vapor mass inside the bubble per unit solid angle, *u* is the speed, ρ is the density, *p* is the pressure, *e* is the specific internal energy, *T* is the temperature, *F* is the energy flux transferred by the thermal conduction mechanism, λ is the thermal conductivity coefficient. The interface between the gas and the fine particle corresponds to the values of *m* = 0 and *r* = 0 while that between the vapor and the liquid fits the equations *m* = *m*_w_ and *r* = *r*_b_. The subscript “0” denotes the values at *t* = 0.

The equation of state and the thermal equation for the vapor, which is considered to be a van der Waals gas, take the following form under the condition of the local thermal dynamics equilibrium:

[3]
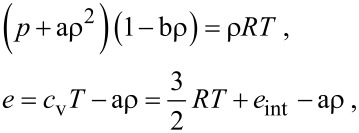


where a and b are constants characterizing the van der Waals gas, *c*_v_ is the specific heat capacity for a constant volume, *e*_int_(*T*) is the energy of the unit of gas mass related to the internal degrees of freedom of molecules, *R* = *k*/*M* is the universal gas constant, *M* is the molecule mass, and *k* is Boltzmann constant.

The liquid temperature variations are determined by the set of thermal conductivity equations:

[4]
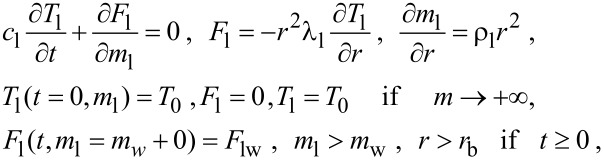


where *m*_l_ is the Lagrangian (mass) coordinate in the region of the presence of the liquid. *T*_l_, *c*_l_(*T*_l_), and λ_l_(*T*_l_) are the temperature, the specific heat capacity, and the coefficient of the thermal conductivity of the liquid, respectively. *F*_lw_ is the energy flux due to the thermal conductivity mechanism.

The distribution of the liquid velocity *u*_l_ over the radius *r* is determined by the continuity equation for incompressible liquid under the condition of spherical symmetry:

[5]
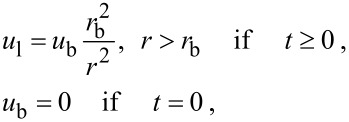


where the pressure *p*_l_ inside the liquid satisfies the Euler equation:

[6]
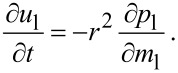


If the temperature *T*_lw_ of the liquid at the edge with the vapor is less than the critical temperature *T*_cr_ of the van der Waals gas then the phase transition takes place at the bubble surface. Considering the edge of the bubble as a hydrodynamical discontinuity (a wave of phase transition) we use the relationships of continuity of the fluxes of mass, momentum, and energy on the surface of the discontinuity:

[7]
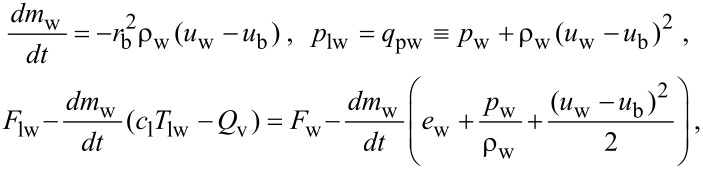


where the subscript “w” characterizes the values on both sides of the wave of phase transition, ρ_w_ << ρ_l_, *p*_lw_ is the pressure on the edge of the bubble, and *Q*_v_(*T*_lw_) is the specific steam heat.

If the liquid temperature at the interface with the vapor is higher than (or equal to) the critical temperature then the phase transition is absent and the relationships in Equation 7 are fulfilled at the interface (*m*_l_ = *m*_w_) under the condition

[8]
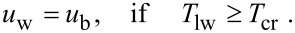


At the surface of the bubble there are two competing processes, namely, surface evaporation and surface condensation. We assume that the vapor is in local thermodynamic equilibrium over the whole volume of the bubble with the exception of the Knudsen layer (of the thickness of several mean free paths of molecules) adjacent to the liquid surface. In this layer, the distribution of the molecules over the speeds can differ substantially from the local equilibrium Maxwell distribution. Since the radius of the bubble is much larger than the mean free path of the gas molecules, the Knudsen layer can be considered as quasistationary thin flat area, which is a part of the wave of phase transformation. Furthermore, since the convective transport dominates over thermal conductivity, the convective flows of mass, momentum and energy are conserved when passing the Knudsen layer under the condition of the violation of the phase equilibrium. The structure of the Knudsen layer and the magnitudes of the gas-dynamics variables in the vicinity of the evaporating surface of the liquid are determined by the equations of the kinetics of phase transitions where we take into account substantially subsonic nature of the flow of gas in the bubble.

## Results

**Figure 2:**
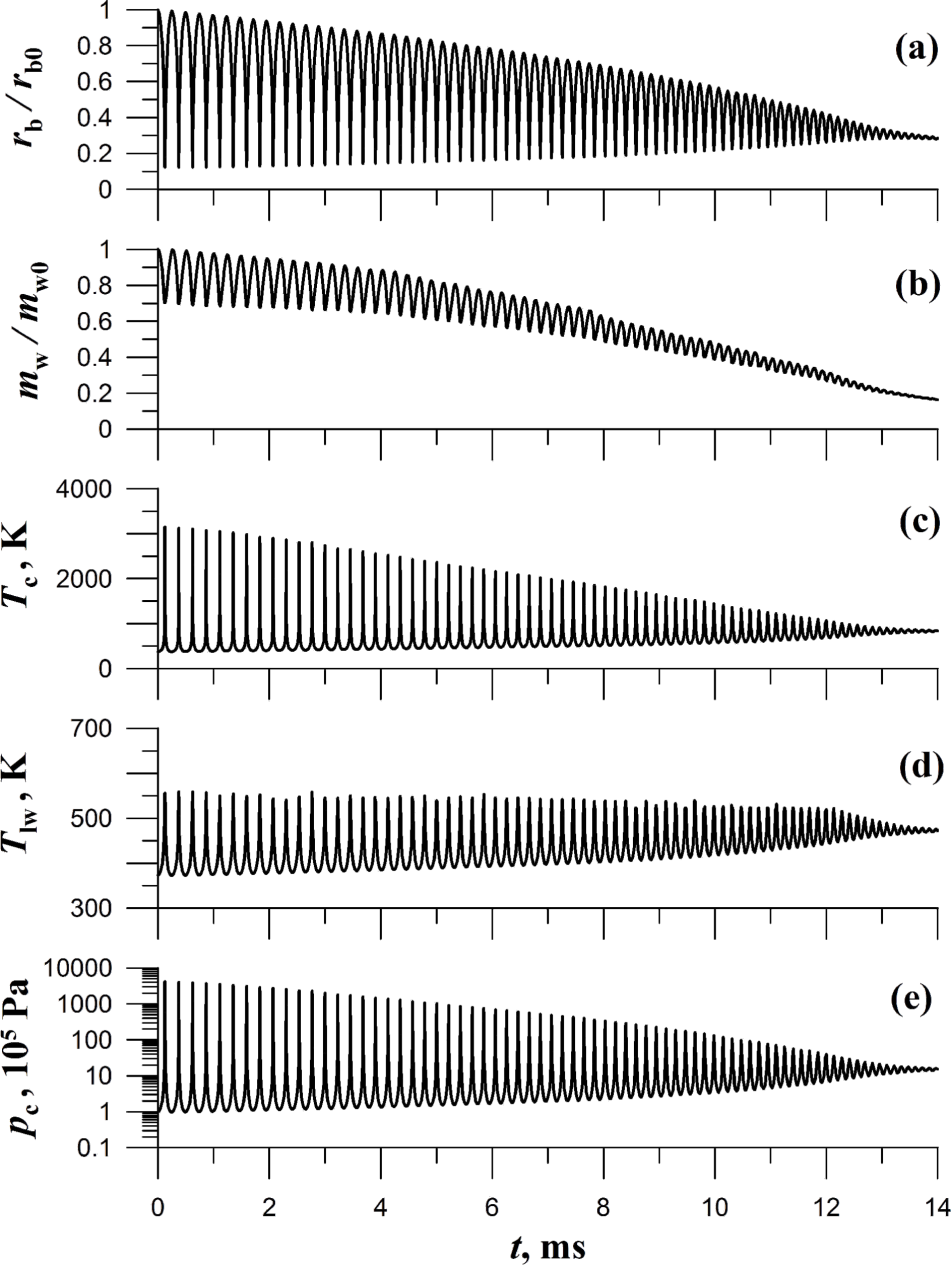
Time dependencies *r*_b_/r_b0_, *m*_w_/*m*_w0_, *T*_c_, *T*_lw_, and *p*_c_ in a pulsating cavitation bubble. Initial parameters are the bubble radius *r*_b0_ = 0.5 cm, the pressure inside the bubble *p*_0_ = 10^5^ Pa, the vapor temperature *T*_0_ = 373 K. The pressure in the liquid far from the bubble is *p*_∞_ = 15*p*_0_.

Here, we present some examples of numerical calculations. We assume that a bubble of the radius *r*_b0_ = 0.5 cm located on a microparticle is in water with the pressure of *p*_0_ = 10^5^ Pa and the temperature of *T*_0_ = 373 K (boiling temperature of water). At the initial moment of time the pressure in the liquid (far from the bubble) increases abruptly to a value of *p*_∞_ = 15 *p*_0_. Figure 2 shows the time-dependence of the following values: the ratio of the radius of the bubble to its initial value *r*_b_/r_b0_, the ratio betwenn the vapor mass inside the bubble and the initial vapor mass *m*_w_/*m*_w0_, the vapor temperature at the border with the solid surface of the microparticle *T*_c_, the liquid temperature at the border of the bubble *T*_lw_; the vapor pressure in the bubble *p*_c_. It is evident that the bubble motion has oscillatory character with successive stages of compression and expansion up to a time *t*_m_ = 14 ms. These oscillations are damped because of heating of the liquid surrounding the bubble. During the first compression of the bubble, its radius and the vapor mass are lowered by factors equal to 8 and 1.4, respectively, while the vapor pressure inside the bubble increases and reaches 4200*p*_0_, the vapor temperature *T*_c_ increases up to the value of 3100 K, which is 1.4 times higher than the corresponding value obtained under the assumptions of the model in [8–11], which takes into account adiabatic compression of the vapor but does not take into account the phase transformation liquid–vapor. Thus the model described in the present paper shows that the cavitation processes occurring on the interface are occasionally more effective than the bulk cavitation processes [8–11].

**Figure 3:**
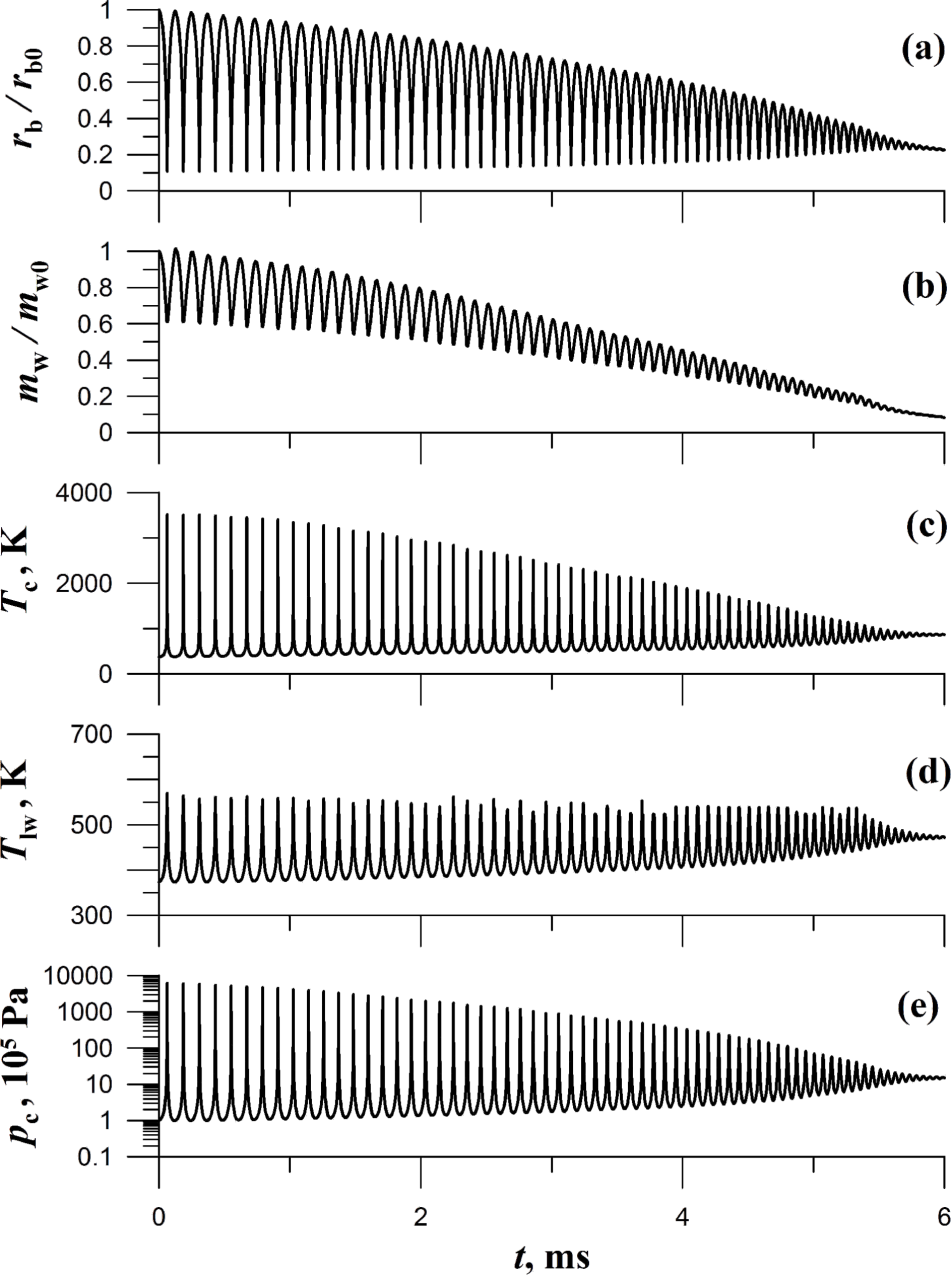
Time dependencies *r*_b_/*r*_b0_, *m*_w_/*m*_w0_, *T*_c_, *T*_lw_, and *p*_c_ in a pulsating cavitation bubble. Initial parameters are the bubble radius *r*_b0_ = 0.25 cm, the pressure inside the bubble *p*_0_ = 10^5^ Pa, the vapor temperature *T*_0_ = 373 K. The pressure in the liquid far from the bubble is *p*_∞_ = 15*p*_0_.

**Figure 4:**
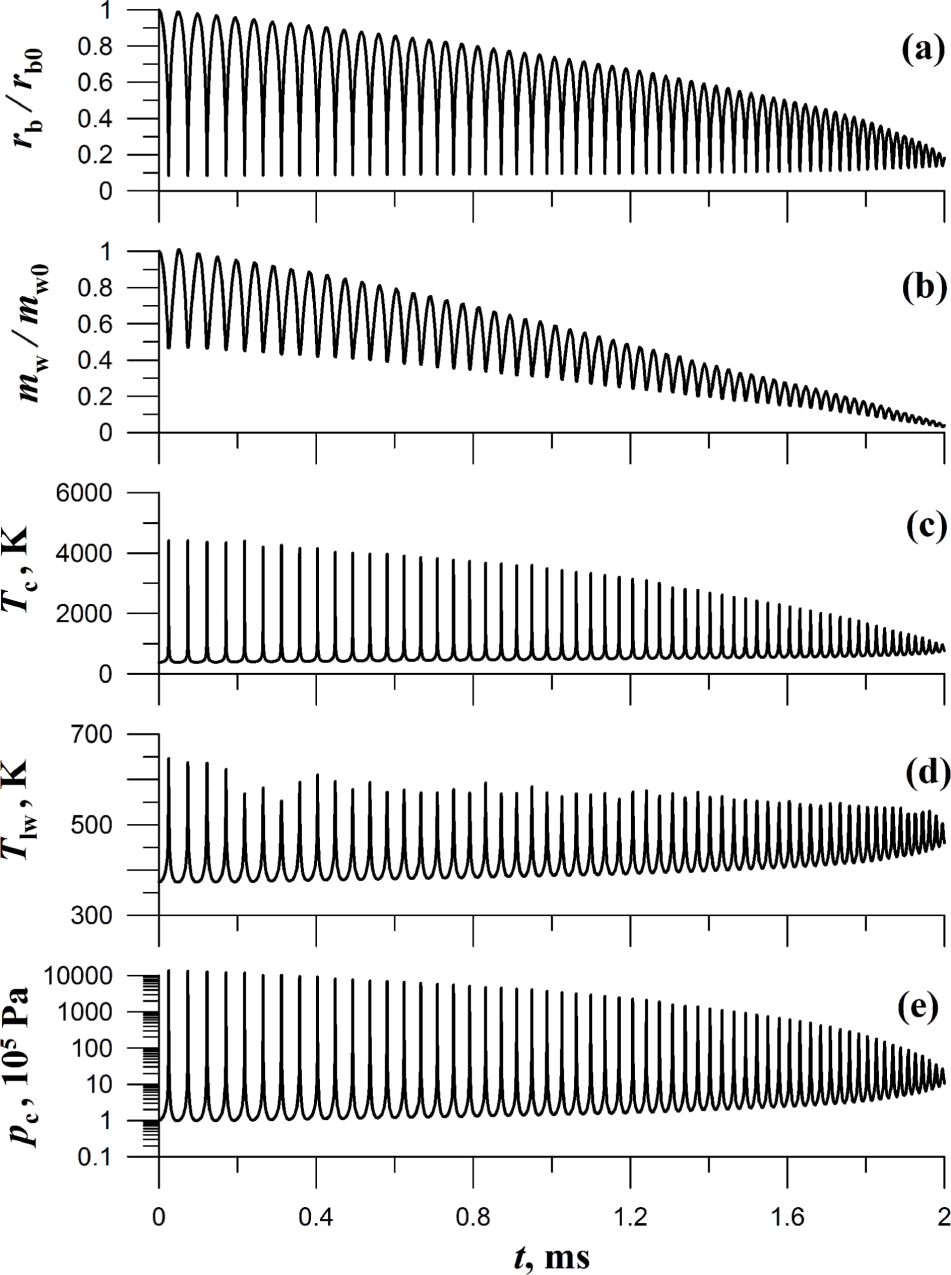
Time dependencies *r*_b_/*r*_b0_, *m*_w_/*m*_w0_, *T*_c_, *T*_lw_, and *p*_c_ in a pulsating cavitation bubble. Initial parameters are the bubble radius *r*_b0_ = 0.1 cm, the pressure inside the bubble *p*_0_ = 10^5^ Pa, the vapor temperature *T*_0_ = 373 K. The pressure in the liquid far from the bubble is *p*_∞_ = 15*p*_0_.

When the initial radius of the cavitation bubble is reduced, the influence of the surface processes, namely condensation, evaporation, and heat transfer between the vapor and liquid, on the bubble dynamics increases, while the pulsation time *t*_m_ decreases. For example, if *r*_b0_ = 0.25 cm (Figure 3) then the pulsation time is *t*_m_ = 6 ms. The vapor mass in the bubble is lowered by a factor equal to 1.6 in the state corresponding to the first maximum compression, the vapor temperature reaching 3500 K, and the vapor pressure in the bubble attaining the magnitude of *p*_c_ = 6250*p*_0_. For *r*_b0_ = 0.1 cm (Figure 4) the pulsation time is *t*_m_ = 2 ms, the first maximum compression fits a 2.1 times decrease in the vapor mass, the temperature increases up to the value of 4400 K, and the vapor pressure grows up to *p*_c_ = 13800*p*_0_.

The microparticle, on which the bubble is located, is melted if the vapor temperature becomes higher than the melting point (*T*_m_)_p_ of the substance of the microparticle, e.g., for quartz (*T*_m_)_p_ = 1883 K, for gold (*T*_m_)_p_ = 1336 K). Each bubble compression results in the extrusion of melt (formed during this compression) out of the bubble. A bubble works like a borer. We calculate the thickness (*X*_mt_)_1_ of the melt which is extruded out of the bubble after the first compression as well as the total melt thickness over during the whole time of the bubble pulsations, *X*_mt_ of the microparticle, i.e., the layer of the microparticle, which is melted and removed out of the particle if the particle size is larger than *X*_mt_. Otherwise, if the particle size is smaller than *X*_mt_ then the particle is melted and, finally, completely destroyed. For the quartz particle we obtain (*X*_mt_)_1_ = 2.6 μm and *X*_mt_ = 62 μm if *r*_b0_ = 0.5 cm, (*X*_mt_)_1_ = 1.9 μm and *X*_mt_ = 60 μm if *r*_b0_ = 0.25 cm, and (*X*_mt_)_1_ = 0.88 μm and *X*_mt_ = 55 μm if *r*_b0_ = 0.1 cm. For the gold particle we have (*X*_mt_)_1_ = 23 μm and *X*_mt_ = 924 μm if *r*_b0_ = 0.5 cm, (*X*_mt_)_1_ = 16 μm and *X*_mt_ = 694 μm if *r*_b0_ = 0.25 cm, and (*X*_mt_)_1_ = 8.8 μm and *X*_mt_ = 465 μm if *r*_b0_ = 0.1 cm.

**Figure 5:**
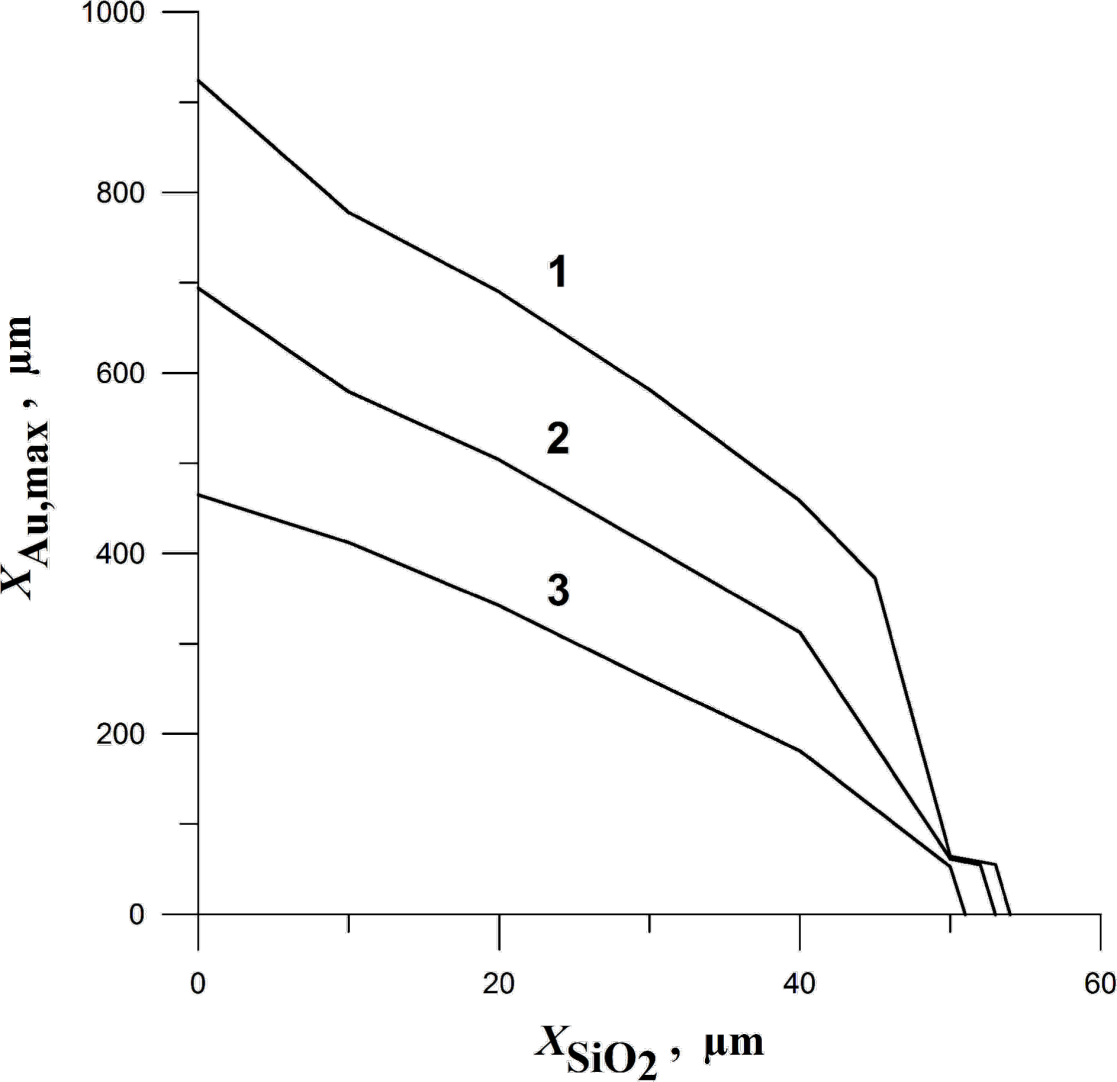
The maximum thickness *X*_Au,max_ of the molten layer of gold versus the thickness *X*_SiO2_ of the quartz layer. Curves 1, 2, and 3 correspond to the initial bubble radii of *r*_b0_ = 0.5 cm, 0.25 cm, and 0.1 cm, respectively. The initial parameters are *p*_0_ = 10^5^ Pa and *T*_0_ = 373 K. The pressure in the liquid far from the bubble is *p*_∞_ = 15*p*_0_.

Consider the situation when a gold particle is located inside a larger quartz particle. Figure 5 shows the dependence of the maximum thickness *X*_Au,max_ of the molten layer of gold on the thickness *X*_SiO2_ of the quartz layer which screens the gold off the heating from the pulsating cavitation bubble. The calculations show that quartz layers with thicknesses above 54 μm completely shield the gold from melting.

## Discussion

In [11] it has been shown that, in the process of melting, the density of gold decreases from ρ_s_ = 19.3 g/cm^3^ to ρ_l_ = 17.31 g/cm^3^, i.e., the volume of the gold particle changes by a factor of ρ_s_/ρ_l_ = 1.12. If the melted particle is incorporated into quartz and its expansion is impossible, its internal pressure will be increased corespondingly. The pressure that arises during the melting of a gold particle incorporated into quartz may be estimated, by using the coefficient of volumetric expansion of gold, *K* = 2.2 Mbar, from the formula

[9]
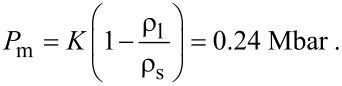


Such a high pressure, several times higher than the strength of quartz (90 kbar), results in the breakup of quartz and separation of the polymineral particle into monomineral fractions. This mechanism works in the case of polymineral nano- and microscale particles considered here when the cavitation processes occur on the interface between liquid and fine solid particles. However, it only occurs for the thicknesses of quartz layer less than 54 μm (Figure 5 which determines the maximum thickness of the molten layer of gold), i.e., when the gold placed inside the quartz particle can be melted.

## Conclusion

The calculations performed in this report show that cavitation melting of microparticles with sizes less than several dozens of micrometers, composed of minerals characteristic of gold deposits, can be realized under shock loading of water. This means, when the pressure in water, which is heated up to the boiling temperature, increases abruptly to a value of *p*_∞_ = 15 bar. As a result of the cavitation melting, the interaction of polymineral microparticles with collapsing cavitation bubbles may lead to breakup and fragmentation of microparticles into monomineral fractions. The cavitation disintegration mechanism can be important in technological processes of beneficiation because it can provide the recovery of disseminated metals from microscale fractions, with sizes of dozens of micrometers, which are obtained when developing the low-grade deposits and reprocessing the ore dumps and tailings.
